# Surgical Diseases of the Abdomen, with Special Reference to Diagnosis

**Published:** 1903-10

**Authors:** 


Surgical Diseases of the Abdomen, With Special Reference to Diagnosis.—By Richard Douglas, M. D., formerly Professor of Gynecology and Abdominal Surgery, Medical Department Vanderbilt University, Nashville, Tenn. P. Blakiston's Son & Co., Philadelphia, Pa.

This most instructive volume of over 800 pages, illustrated by 20 full page plates, deserves a place in every physician's and surgeon's library.

The author's eighteen years of experience as a teacher and practical worker in the special field of abdominal surgery has admirably fitted him for authorship. This work from a diagnostic standpoint is specially valuable to the physician, and, from an operative and post-operative standpoint is valuable to the surgeon.

The chapter on appendicitis alone is worth the price of the work. The chapter on gall stones is specially interesting. The schema on differential diagnosis and the method of arranging same brings out all the confusing points so clearly that one could hardly make a mistake. On the whole the book is exceedingly well gotten up—presented to its readers in choice, descriptive language.

The illustrations are all good, most of them of special value on account of their bearing on relational anatomy. R. & L.

